# The Memorial to Sir Andrew Clark

**Published:** 1894-05-12

**Authors:** W. E. Gladstone


### The Memorial to Sir Andrew Clark.

By The Right Hon. W. E. Gladstone, M.P., and "Punch."

Mr. Gladstone delivered the following speech at the Andrew Clark Memorial meeting held in Princes' Hall on Thursday, May 3rd:

May it please your Royal Highness—I will make no apology, after the words that have fallen from your Royal Highness, for availing myself of the permission which has been kindly granted to me to say, without rising from my seat, a few words that it may become me to say on the present occasion. I am very glad indeed, not for the first time, nor the second—nor, perhaps, the tenth or twentieth time—to meet for a great public object under your Royal Highness's auspices, and I will say with great confidence that I do not believe you ever gave the advantage of your countenance and your high position to a worthier cause than that which has brought us together to-day. For my own part, Sir, if it be with some small effort that I have come here to bear my humble part in the proceedings of the day, I must say that to have been absent from them, to have missed the opportunity of bearing my testimony to the noble life of Sir Andrew Clark, would have been a standing grief and mortification. I have the honour to address an audience which is composed, I have no doubt, in no inconsiderable degree, of Sir Andrew Clark's brethren in the noble profession to which he belonged—(cheers)—and in truth, although Sir Andrew Clark himself was a person whose name constitutes in itself an ample warrant and apology for a large gathering such as that I see before me, yet I feel also that this occasion is one which associates itself with the profession at large. Undoubtedly it was to me a matter of the most lively satisfaction when I observed, as the whole world must have observed, on the death of Sir Andrew Clark, the thrill of affectionate interest which ran through the whole of that great profession, the sense of the heavy loss they had sustained, and the desire to render some worthy and adequate testimony to such a memory as that of Sir Andrew Clark. The profession itself is one with regard to which it is impossible think, not to be conscious that, great as has been its position in our generation, and in some generations previous, it is a position continually advancing and continually rising. In truth, if we go back for some distance, the other learned professions, as they are sometimes called, undoubtedly had the start of the medical profession. I think that as much as 400 or 500 years ago, in the very first beginnings of the commerce of the country, the lawyers in scores and hundreds sprang up almost like mushrooms in the ground, to dispose of disputes which necessarily arise wherever property has been brought into existence. At that time medical men were hardly heard of. But the position of the profession at the present day has become one of vital and commanding interest to the whole of society, and I anticipate that that interest must continue. While wealth increases, while inventions and discoveries increase, wants will increase, enjoyments will increase, and in connection with wants and enjoyments there will, I fear, be a corresponding development of infirmity and of disease, and the medical profession braces itself to grapple with the situation which has been created, and continually advances in knowledge and in competence. My own life is long enough to have enabled me to witness, and in some degree to measure, the change which has taken place. I have had the good fortune of knowing many distinguished men of the profession during the last three score years; but I have also seen a great change in the capacity, in the attainments, in the competency to deal with the difficult and almost insoluble problems that are continually presenting themselves to the minds of medical men. It appears to me that it was eminently desirable that in a time like this a man such as Sir Andrew Clark should rise to the head of the profession, for, after all, we require something more than knowledge, something more than skill. We require a great devotion to the purpose of the profession, and that devotion never, I think, was exemplified in a more remarkable manner than in the career of Sir Andrew Clark. I remember a very small incident. When Sir Andrew Clark had been spending his well-earned vacation at a residence which he temporarily occupied in Scotland, and when the few weeks that he allowed himself for retirement were nearly exhausted, a friend came to him, and supposed that he was paying him a compliment by saying, "I condole with you, Sir Andrew Clark, on the approaching close of your vacation." Sir Andrew Clark looked at him and said, "I love my profession." He did love his profession. His whole heart and soul were engaged in that profession. He loved it not only with a sincere and cordial love, but with a chivalrous devotion. We need not say that the age of chivalry has altogether passed since we have, and I trust we may still have among us men of the type of Sir Andrew Clark. (Cheers.) There was no part of him, no faculty, no portion of his time which he did not devote, with his whole soul and the whole strength of purpose that he could command, to the great work that was before him, not merely in the attainment of knowledge, but in the application of that knowledge to the diminution of human suffering. I think the profession, if I may presume to say so, has done well in determining that Sir Andrew Clark might in the present age be taken up by common consent as a typical man, representative of all that is best and noblest in the profession and in its purposes. In every profession there is something more—I would almost say, perhaps, something more important—required than knowledge and capacity, and that is character. The position of medical men is one which brings them more and more into the confidence of those for whose benefit they consult; and in order that they may be worthy of that confidence, and may turn it to account, it is not requisite merely that they shall have studied with vitality, and even with ardour, the difficult subjects to which they devote their minds, but it is that all the qualities that give force and weight to character shall be exhibited by them in conjunction with their professional skill. I hope that our proceedings will be read extensively throughout the country, and especially I am quite certain that they will be everywhere read by members of the medical profession. Of those, some are advanced in life, but many also are very young, and I rejoice to think what an honour, benefit, and privilege it is to the young members of the profession to be called upon to contemplate a career and a character like those of Sir Andrew Clark. Dr. Church has given us the advantage, in a tract which has recently been published, of a most interesting sketch of that character. He has shown how, in the case of Sir Andrew Clark, the boy was father of the man. He tells us that in his very early days, when he was the merest youth in the town of Dundee, in the very first stage, the initial stage, of his profession, how at that time he acquired first the deep love of that profession; secondly, that profound sense of religion which attended him through his whole life, which supplied him with its guiding principle, and which produced in him this singular result, that when his mind had been occupied and absorbed in the arduous questions continually brought before him in the course of his practice, it was his delight, and all those who knew him well, I am sure, would sustain me in what I say, it was his delight and his practice to find recreation, not in trifles and in frivolities, but in betaking himself naturally to the consideration of divine things. It would be great impertinence and presumption on my part if, in moving this resolution, I were not to observe what is the portion of it to which I am in any degree competent to speak. The resolution is: "That, in recognition of the great services rendered to the community by the late Sir Andrew Clark, Bart., M.D., a memorial be established which shall perpetuate his name and his work." It is not for me to give opinions upon the medical eminence and skill of Sir Andrew Clark. I may record my sense of the benefits that I have derived from them. I may say that I have come here to fulfil the office, and to pay the tribute, not only of duty and of respect, but of gratitude and of personal affection. Of course, I am not competent to judge of the medical skill of Sir Andrew Clark. What I have seen are his patience, his thoroughness, his absorption in the case of his patient, as if that one case was the single subject with which he had to occupy his mind. And I must add in my own instance that I have also had to note the warmth of friendship, the assiduous prosecution of the task that he had set to himself of watching my health, which I know not how adequately to describe. But I may say this, that, although he was much younger than I am, yet he followed me from month to month and week to week, and never more than in the last year of his existence, with something that resembled a paternal affection. It is good for us to look back upon a life of this kind, and in my own case the recollections which I cherish, and ever shall cherish during the residue of my life, reach over a period of nearly thirty years. It was the happy lot of my wife to be among the first to recognise what was in Sir Andrew Clark, for she had at that period become personally acquainted with the case of the London Hospital, and when she came back from one of her first visits, describing what she had seen and the persons with whom she had become acquainted, she at that early date described to me among them him whom she called "that great Andrew Clark." Those words, gentlemen, have been verified by a long course of subsequent experience, and I trust that the results of this meeting will show that Sir Andrew Clark is not forgotten, and that there is a desire on the part of the profession, on the part of that large number of persons who profited by his skill and care, and on the part of many members of the community at large, that such a name shall be commemorated among us. Sure I am that whatever happens, whatever may have been the past advances of the medical profession, and they are great, whatever may be the future advances of that profession, and in all likelihood they will be greater still, there never will come a time when that profession will not be justly satisfied and glad to have recorded upon its annals a name such as the name of Sir Andrew Clark.

